# Targeting the nuclear export receptor exportin‐1 in acute myeloid leukaemia: From biology to clinical translation

**DOI:** 10.1002/ctm2.70676

**Published:** 2026-05-03

**Authors:** Yifan Liu, Xiaoya Yun, Weidong Ding, Suxiao Li, Hui Liu

**Affiliations:** ^1^ Department of Hematology, Beijing Hospital, National Center of Gerontology, Institute of Geriatric Medicine Chinese Academy of Medical Sciences & Peking Union Medical College, National Center for Clinical Laboratories, Chinese Academy of Medical Sciences & Peking Union Medical College Beijing China

**Keywords:** acute myeloid leukaemia, exportin‐1, selective inhibitors of nuclear export, venetoclax‐based combinations, XPO1 inhibition

## Abstract

**Background:**

Exportin‐1 (XPO1), a key regulator of nucleocytoplasmic transport, is frequently dysregulated in acute myeloid leukemia (AML) and contributes to leukemogenesis, disease progression and therapeutic resistance. Selective inhibitors of nuclear export (SINEs), especially selinexor and eltanexor, have shown promising antileukemic potential. However, their clinical value, optimal therapeutic positioning and rational use in AML remain to be fully clarified.

**Methods:**

We collected and reviewed relevant literature to summarize the biological roles of XPO1 in AML and the therapeutic potential of XPO1 inhibitors in preclinical and clinical settings.

**Results:**

In this review, we focus on the nuclear export function of XPO1 and its pathogenic role in AML. We summarize the mechanisms of action, preclinical evidence, clinical trial results, adverse effects, resistance mechanisms and potential response biomarkers associated with XPO1 inhibitors in AML.

**Conclusions:**

XPO1 inhibition has emerged as a promising therapeutic strategy for AML, offering a novel approach to targeting aberrant nucleocytoplasmic transport and overcoming treatment resistance. Future studies should focus on optimizing dosing schedules, identifying predictive biomarkers and developing effective combination strategies in molecularly selected AML populations.

**Key Points:**

1. XPO1 hyperactivation rewires nucleocytoplasmic transport and sustains leukaemogenic programs in genetically defined acute myeloid leukaemia (AML) subsets.
2. Selective XPO1 inhibitors (selinexor, eltanexor) show preferential activity in NPM1‐mutated, DEK::NUP214‐positive and SF3B1‐mutated myeloid neoplasms.
3. Combination strategies with hypomethylating agents, BCL‐2 inhibitors and other targeted therapies enhance depth and durability of responses but are limited by toxicity.
4. Future clinical trials should focus on molecularly selected populations, biomarker‐guided dosing and translational endpoints such as measurable residual disease (MRD) and clonal dynamics.

## Graphical abstract

• XPO1 hyperactivation promotes leukaemogenesis by altering nucleocytoplasmic transport and transcriptional control in acute myeloid leukaemia (AML).

• Selinexor and eltanexor show preferential activity in NPM1‐mutated, DEK::NUP214‐positive and SF3B1‐mutated myeloid neoplasms.

• Combination strategies with hypomethylating agents, venetoclax and other targeted therapies may improve response depth and durability but remain toxicity‐limited.

• Future trials should emphasise molecular selection, biomarker‐guided dosing, measurable residual disease (MRD) monitoring and clonal evolution.

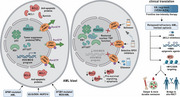

## 1 INTRODUCTION

Acute myeloid leukaemia (AML) is characterised by high rates of relapse and treatment resistance, and long‐term remission and survival remain particularly limited in older patients and those with multiple comorbidities.^1^, ^2^ Over the past decade, advances in molecular classification, targeted therapies and immunotherapies have substantially improved the precision and individualisation of AML treatment.^3^, ^4^, ^5^ Among these, the low‐intensity combination of the BCL‐2 inhibitor venetoclax (VEN) with the hypomethylating agent azacitidine (AZA; the VEN‐AZA, or VA, regimen) has emerged as a new treatment paradigm and is now recommended by major international guidelines (including NCCN, ELN and ESMO) as a first‐line option for older patients or those unfit for intensive chemotherapy.^6^, ^7^, ^8^, ^9^, ^10^ Randomised trials and real‐world cohorts have shown that the VA regimen markedly increases complete remission (CR) and CR with incomplete haematologic recovery (CRi) rates and prolongs overall survival in these populations.^6^, ^7^, ^11^ Nevertheless, large multicentre studies have also shown that salvage options after VA failure are extremely limited, with median survival typically less than 1 year overall and often less than 6 months once relapse has occurred.^12^, ^13^, ^14^ Consistent with these observations, our own single‐centre Chinese real‐world cohort showed high initial CR/CRi rates but a median event‐free survival (EFS) of approximately 9.9 months, with most patients still experiencing disease progression within the first year.^15^ Accordingly, a central challenge in contemporary AML practice is how to rationally integrate therapies with genuinely novel mechanisms of action into existing standards of care to deepen remission, prolong its duration, and create an optimal window for subsequent haematopoietic stem cell transplantation.

Aberrant nucleocytoplasmic transport is recognised as an important pathogenic mechanism in the development and progression of AML.^16^, ^17^ Exportin‐1 (XPO1), also known as chromosome region maintenance protein 1 (CRM1), is the principal nuclear export receptor and mediates nuclear export of a broad range of tumour suppressor proteins and cancer‐related signalling mediators, including p53, FOXO and NPM1.^18^, ^19^ Hyperactivation of XPO1 leads to cytoplasmic mislocalisation of tumour suppressors, sustained activation of oncogenic transcriptional programs and, through multilayered rewiring of transcriptional, epigenetic and translational networks, promotes leukaemic cell proliferation, apoptosis evasion and clonal evolution.^18^, ^20^ Selective inhibitors of nuclear export (SINEs) targeting this nodal transporter covalently inhibit XPO1 activity and restore the nuclear localisation and function of tumour suppressor pathways.^20^ Selinexor, the first XPO1‐targeted SINE to enter clinical use, has been approved for the treatment of relapsed or refractory multiple myeloma and diffuse large B‐cell lymphoma; however, its optimal therapeutic positioning, beneficiary subgroups and rational combination strategies in AML are still under active investigation.^18^, ^21^, ^22^

In this review, we first summarise the biological functions of XPO1 in AML and the mechanisms by which dysregulated nuclear export contributes to leukaemogenesis and disease progression, with particular emphasis on specific molecular subtypes. We then review recent preclinical and clinical advances with XPO1‐targeted SINE compounds in AML, focusing on both genotype‐defined subsets and combinations with hypomethylating agents, BCL‐2 inhibitors and other pathway‐directed therapies. This review was informed primarily by representative studies on XPO1 inhibitors in AML published over the past 5 years and retrieved from PubMed and Web of Science, together with relevant clinical trial registry records. Finally, informed by available toxicity data and emerging insights into resistance, we discuss key challenges for clinical implementation and outline future directions for integrating XPO1‐targeted strategies into risk‐adapted stratification and individualised treatment algorithms for patients with biologically and clinically complex AML.

## 2 BIOLOGICAL ROLES OF THE NUCLEAR EXPORT RECEPTOR EXPORTIN‐1 IN AML

**FIGURE 1:**
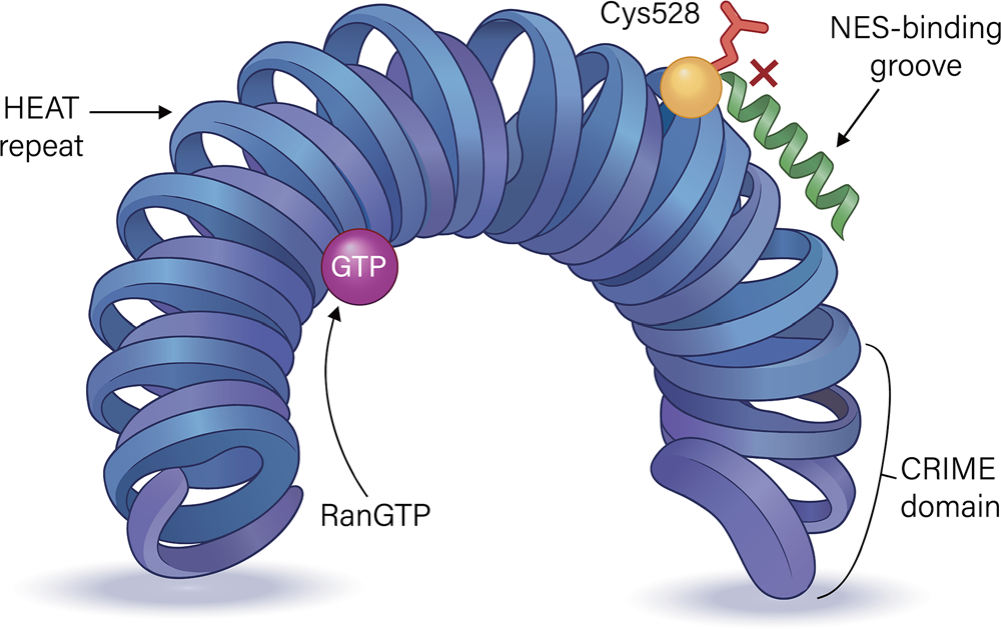
Structural organization of the nuclear export receptor XPO1 and the NES‐binding cleft targeted by SINE compounds. XPO1 consists of 21 tandem HEAT repeats that assemble into a toroidal, ring‐like architecture, with a hydrophobic NES‐binding cleft located on the convex outer surface. Binding of RanGTP stabilizes an open conformation that permits loading of NES‐containing cargo proteins. The non‐catalytic residue Cys528, located at the base of the NES‐binding cleft, is covalently targeted by selective inhibitors of nuclear export (SINEs), which occupy the groove and sterically prevent NES binding. The N‐terminal CRIME domain is indicated.

Nuclear export proteins, particularly the karyopherin exportin‐1 (XPO1), play a central role in maintaining cellular homeostasis by regulating nucleocytoplasmic transport and thereby controlling the subcellular distribution of numerous proteins and RNAs.^18^ In AML, XPO1 overexpression or dysregulated XPO1‐dependent nuclear export alters the localisation of key tumour suppressor proteins and signalling mediators, disrupts normal regulatory networks and promotes leukaemogenesis and disease progression.^16^ Structurally, XPO1 is a ∼123‐kDa karyopherin‐β family protein composed of 21 tandem HEAT repeats arranged into a toroidal, ring‐like architecture, with an N‐terminal CRIME domain involved in RanGTP binding (Figure 1).^23^, ^24^ A highly conserved hydrophobic groove on the convex outer surface of the HEAT repeats forms the NES‐binding cleft, which accommodates leucine‐rich nuclear export signals (NESs) on cargo proteins.^25^ Binding of RanGTP stabilises an ‘open’ conformation that favours cooperative loading of NES‐containing tumour suppressor proteins and oncoproteins.^26^, ^27^ SINE compounds, such as selinexor and eltanexor, exploit this structural vulnerability by covalently modifying the non‐catalytic cysteine residue Cys528 at the base of the NES‐binding cleft, thereby blocking NES binding, functionally inhibiting XPO1‐mediated nuclear export and promoting nuclear retention of key tumour suppressor protein.^28^, ^29^

### 2.1 XPO1 and the nucleocytoplasmic transport machinery

XPO1 is one of the principal nuclear export receptors, responsible for exporting hundreds of proteins and multiple classes of RNA from the nucleus to the cytoplasm in a Ran GTPase‐dependent manner.^30^ In the nucleus, Ran predominantly exists in its GTP‐bound form (RanGTP).^31^ XPO1 recognises NES‐bearing cargo proteins and, together with RanGTP, forms a trimeric export complex.^27^ This complex interacts with nucleoporins within the nuclear pore complex (NPC) and traverses the NPC into the cytoplasm, where RanGTP is hydrolysed to RanGDP under the control of Ran GTPase‐activating protein (RanGAP) and Ran‐binding proteins (RanBP1/2). Hydrolysis triggers dissociation of the trimeric complex, release of the cargo and recycling of XPO1 back to the nucleus for another round of transport.^32^ The steep RanGTP gradient across the nuclear envelope is essential for the directionality and fidelity of nuclear export.^33^

**FIGURE 2:**
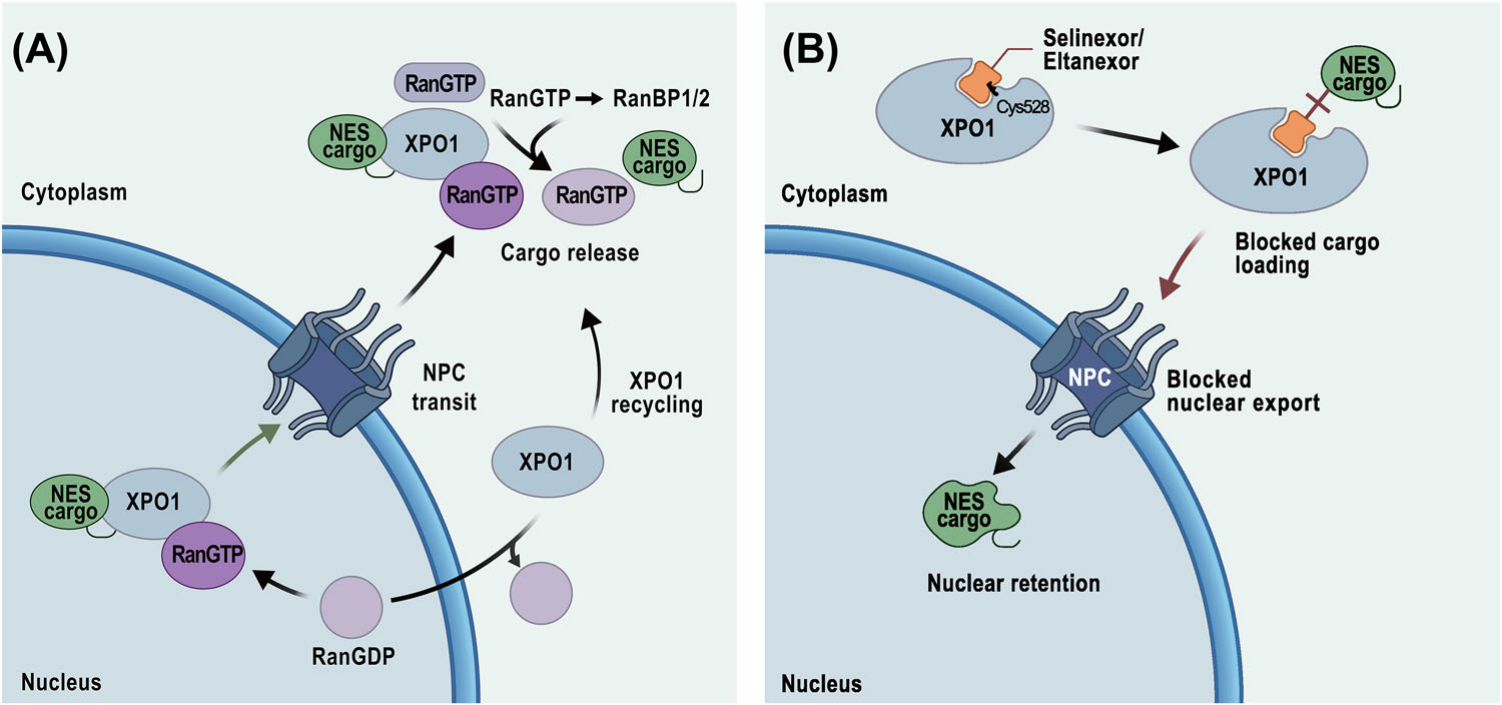
XPO1‐mediated nuclear export and its inhibition by selective inhibitors of nuclear export (SINEs). (A) Under physiological conditions, XPO1 recognizes leucine‐rich nuclear export signal (NES)‐containing cargo proteins and, together with RanGTP, forms a trimeric export complex in the nucleus. This complex translocates through the nuclear pore complex (NPC) into the cytoplasm, where RanGTP hydrolysis, facilitated by RanGAP and RanBP1/2, triggers complex disassembly and cargo release. XPO1 is subsequently recycled back to the nucleus, and the RanGTP gradient across the nuclear envelope ensures the directionality of nuclear export. (B) Selective inhibitors of nuclear export (SINEs), such as selinexor and eltanexor, covalently bind to the Cys528 residue within the NES‐binding groove of XPO1, thereby preventing cargo loading and blocking nuclear export. As a result, NES‐containing cargo proteins are retained in the nucleus.

Beyond its role in interphase nucleocytoplasmic transport, XPO1 localises to kinetochores and centrosomes during mitosis and contributes to microtubule nucleation, centrosome integrity and spindle assembly, functions that are at least partly independent of its canonical export activity.^34^ In AML cells, particularly those with high proliferative and metabolic activity, XPO1 is often upregulated, thereby enhancing the nuclear export of cargoes involved in cell‐cycle control, DNA repair and apoptosis evasion (Figure 2).^18^, ^35^

### 2.2 Dysregulation of XPO1 in AML

Across AML cohorts, XPO1 is frequently overexpressed, and high XPO1 levels are associated with adverse prognosis and treatment resistance. The mechanisms driving XPO1 upregulation are not fully elucidated, but existing data suggest that common lesions such as MYC overexpression and TP53 dysfunction can transcriptionally promote XPO1 expression.^36^ Somatic mutations within the NES‐binding groove (e.g., E571K) can modify the conformational state and cargo recognition profile of XPO1.^37^ Although such mutations are more prevalent in B‐cell malignancies and Hodgkin lymphoma, similar structural alterations have only rarely been described in AML, and their functional relevance in this disease remains uncertain.^38^ Although naturally occurring XPO1 mutations appear to be uncommon in AML, their biologic relevance may extend beyond simple mutational annotation. At present, however, the functional and therapeutic significance of individual XPO1 mutations in AML remains insufficiently defined, and these lesions are best viewed as mechanistic clues that may inform future studies of XPO1 dependency and treatment adaptation.

In selected molecular subtypes, the interplay between XPO1 dysregulation and leukaemia‐defining genetic lesions is particularly prominent.^39^ In NPM1‐mutated AML, mutant NPM1 (NPM1c) acquires a novel NES motif and becomes dependent on XPO1‐mediated aberrant cytoplasmic export.^38^ This process relocalises the nuclear transcription factor PU.1 and its associated partners to the cytoplasm, disrupts differentiation‐related transcriptional networks, and sustains a leukaemia stem cell‐like state.^39^ In high‐risk molecular subsets such as FLT3‐ITD‐positive AML, XPO1 promotes nuclear export of pro‐apoptotic factors including p53 and FOXO3A, attenuating nuclear apoptotic signalling and cooperating with oncogenic kinase pathways to enhance cell survival.^40^

Beyond XPO1 itself, related components of the export machinery such as Ran, eIF4E, HuR and LRPPRC are upregulated in several haematologic malignancies, including subsets of AML, thereby amplifying nuclear export imbalance and contributing to molecular heterogeneity and resistance.^41^

### 2.3 Key XPO1 cargoes relevant to AML

XPO1 mediates the nuclear export of a wide spectrum of proteins and RNAs, and aberrant export of these cargoes plays a critical role in AML pathogenesis and progression.^42^ Broadly, XPO1 cargoes can be divided into two major categories. The first comprises tumour suppressor proteins, such as p53, RB1, FOXO1/FOXO3A, APC, BRCA1/2, p21 and p27. Cytoplasmic sequestration of these proteins leads to loss of their nuclear functions, thereby promoting uncontrolled proliferation and resistance to apoptosis.^41^ The second category includes oncogenic proteins and signalling regulators whose aberrant nucleocytoplasmic distribution may support malignant behaviour, such as cyclin B1, cyclin D1, SNAIL, TERT, survivin, topoisomerase IIα (TOP2A), c‐ABL and YAP1.^43^, ^44^ Aberrant cytoplasmic accumulation of these oncogenic cargoes augments downstream transforming signals and supports leukaemic cell growth and survival.^45^

In haematologic malignancies including AML, XPO1 also cooperates with the eIF4E–LRPPRC complex to enhance nuclear export and translation of specific oncogenic mRNAs.^46^ eIF4E recognises transcripts containing an eIF4E sensitivity element (4ESE) and, through LRPPRC and XPO1, mediates their selective export.^47^ These transcripts include key anti‐apoptotic factors such as BCL2 and MCL1, and their enhanced export and translation markedly reinforce cytoplasmic pro‐survival signalling. MDM2 is another XPO1 cargo, and its nuclear export further dampens p53‐mediated tumour suppressor activity. Moreover, CRM1‐dependent nuclear export of TOP2A has been linked to resistance to anthracycline‐based chemotherapy in several tumour models.

In addition to proteins, XPO1 exports multiple RNA species and ribonucleoprotein (RNP) complexes, including preribosomal RNPs involved in ribosome biogenesis, U snRNAs required for pre‐mRNA splicing and selected cancer‐related mRNAs controlled by the eIF4E–LRPPRC–XPO1 axis.^48^ In SF3B1‐mutated myelodysplastic syndromes (MDSs)/AML, preliminary data suggest that such clones might be particularly sensitive to XPO1 inhibition, although the direct molecular links between XPO1‐mediated RNA export and spliceosome dysfunction remain to be fully delineated.^49^, ^50^

### 2.4 Contribution of XPO1 to leukaemogenesis and AML pathobiology

In AML, hyperactivation of XPO1 promotes leukaemogenesis and disease maintenance by functionally inactivating nuclear tumour suppressor proteins and driving cytoplasmic accumulation of oncogenic cargoes.^51^ In NPM1‐mutated AML, XPO1‐mediated export of NPM1c and its partner PU.1 alters the composition and localisation of nuclear transcriptional complexes.^52^ As a result, complexes involving PU.1, CEBPA and RUNX1 are misdirected, leading to repression of differentiation‐associated genes and sustained activation of HOX/MEIS1 programs, thereby reinforcing leukaemia stem cell‐like properties.^53^ In high‐risk molecular subsets, including FLT3‐mutated AML, XPO1‐dependent nuclear export of pro‐apoptotic factors attenuates nuclear death signalling and cooperates with oncogenic kinase‐driven proliferative pathways to promote leukaemic cell survival and expansion.^54^

Beyond such gene‐specific effects, dysregulated XPO1 activity can drive epigenetic perturbations, enhance ribosome biogenesis and augment translation of oncogenic mRNAs, thereby sustaining molecular heterogeneity and drug‐resistant phenotypes in AML at multiple regulatory levels.^55^ Preclinical studies have shown that XPO1 inhibition restores nuclear localisation and function of multiple tumour suppressor pathways, induces apoptosis and differentiation of leukaemic cells (e.g., promoting monocytic differentiation in NPM1‐mutated AML) and can act through both p53‐dependent and partially p53‐independent mechanisms.^56^ In AML cohorts, intact TP53 function has been associated in some studies with greater sensitivity to selinexor.

**FIGURE 3:**
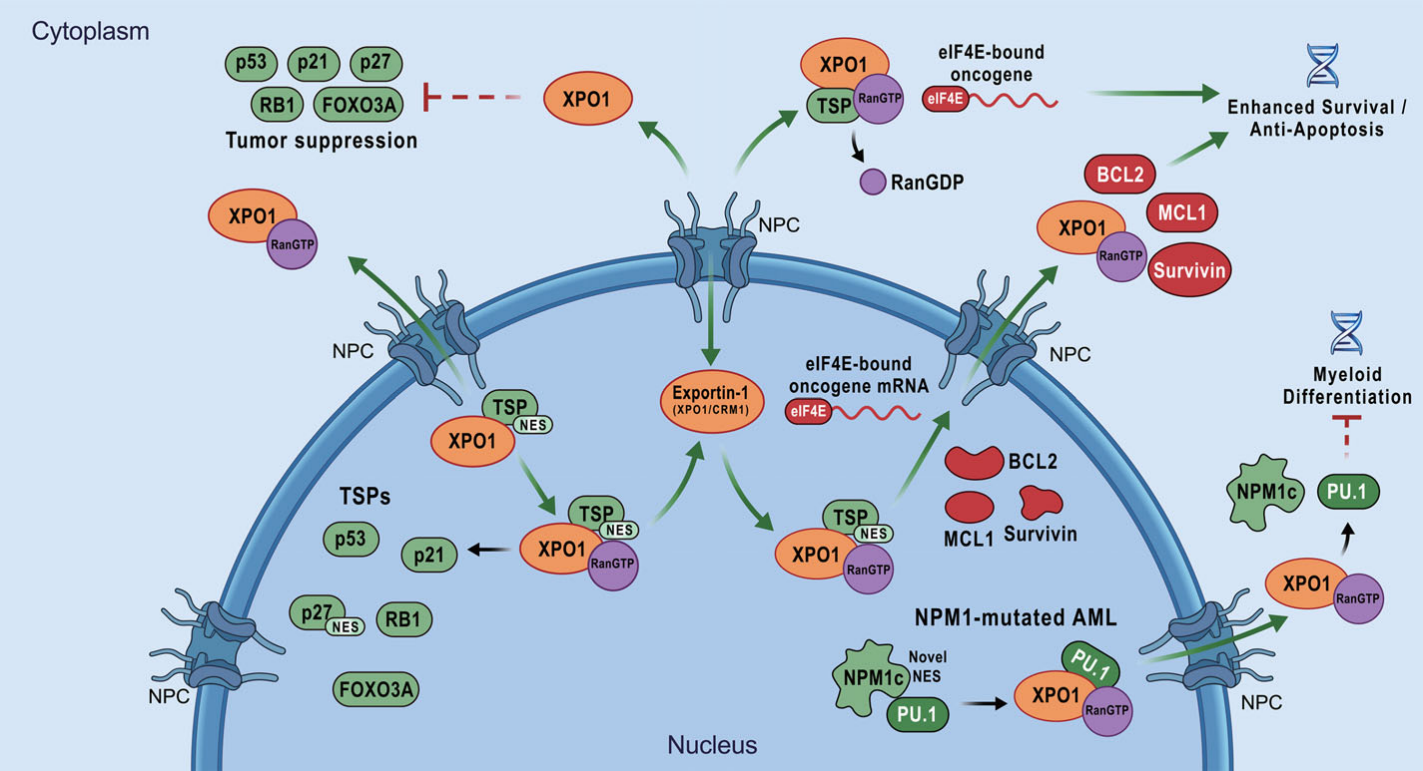
Pathogenic roles of XPO1‐mediated nuclear export in AML. In AML, hyperactivation of XPO1 promotes nuclear export and cytoplasmic sequestration of multiple tumor suppressor proteins (TSPs), thereby weakening tumor‐suppressive signaling and contributing to leukemogenesis. XPO1‐mediated export also enhances the cytoplasmic accumulation of pro‐survival proteins and cancer‐related transcripts, thereby reinforcing survival signaling. In NPM1‐mutated disease, mutant NPM1 (NPM1c) and its transcriptional partner PU.1 are mislocalised to the cytoplasm in an XPO1‐dependent manner, leading to repression of differentiation‐associated genes and sustained activation of HOX/MEIS programs, which reinforce leukemia stem cell like properties. In parallel, XPO1 cooperates with eIF4E‐associated export programs to enhance nuclear export and translation of oncogenic mRNAs, including BCL2 and MCL1, thereby amplifying pro‐survival signaling. Through these convergent effects on transcriptional control, apoptosis regulation and translational output, dysregulated XPO1 acts as a central node in leukemogenesis and disease maintenance in AML.

Collectively, these findings identify XPO1 as a major regulator of transcriptional control, apoptotic signalling, epigenetic regulation and translational programs in AML, supporting its central role in leukaemia initiation and maintenance (Figure 3).

### 2.5 Genetic evidence for the target‐specific role of XPO1 in AML

At present, direct evidence based on XPO1 knockout or genetic ablation in AML models remains relatively limited, but supportive findings have emerged in several molecularly defined subtypes. Charles Cano et al. showed in DEK::NUP214‐positive AML models that deletion of XPO1 markedly impaired leukaemic cell survival and induced cell‐cycle arrest and apoptosis; in patient‐derived xenograft models, XPO1 inhibition also delayed disease progression and reduced leukaemic burden. Further mechanistic analyses showed that XPO1 co‐localises with DEK::NUP214 on chromatin, whereas XPO1 loss or pharmacologic inhibition disrupts this aberrant transcriptional state and downregulates programs associated with cell‐cycle progression and self‐renewal.^57^ Collectively, these findings indicate that, at least in selected molecular AML subtypes, XPO1 is not merely a drug‐binding target but a functional node required for maintenance of the leukaemic state.

In addition to direct genetic ablation studies, another important line of evidence supporting the target specificity of XPO1 derives from genetic on‐target validation. Neggers et al. demonstrated using a gene‐editing strategy that alteration of the critical drug‐binding residue Cys528 in XPO1 conferred marked resistance to selinexor and attenuated the canonical phenotype of nuclear export inhibition.^58^ These findings provide genetic evidence that the core cellular effects of SINE compounds are highly dependent on engagement of a specific residue within XPO1.^59^ In AML, this provides important orthogonal support for attributing the anti‐leukaemic effects of selinexor and eltanexor to on‐target XPO1 inhibition and further strengthens the mechanistic interpretation of related preclinical studies.

Taken together, these findings support the view that XPO1 is not merely a broadly druggable nuclear export factor, but a genetically and mechanistically validated leukaemia‐maintenance dependency in defined molecular contexts.

## 3 PRECLINICAL STUDIES OF XPO1 INHIBITION IN AML

**FIGURE 4:**
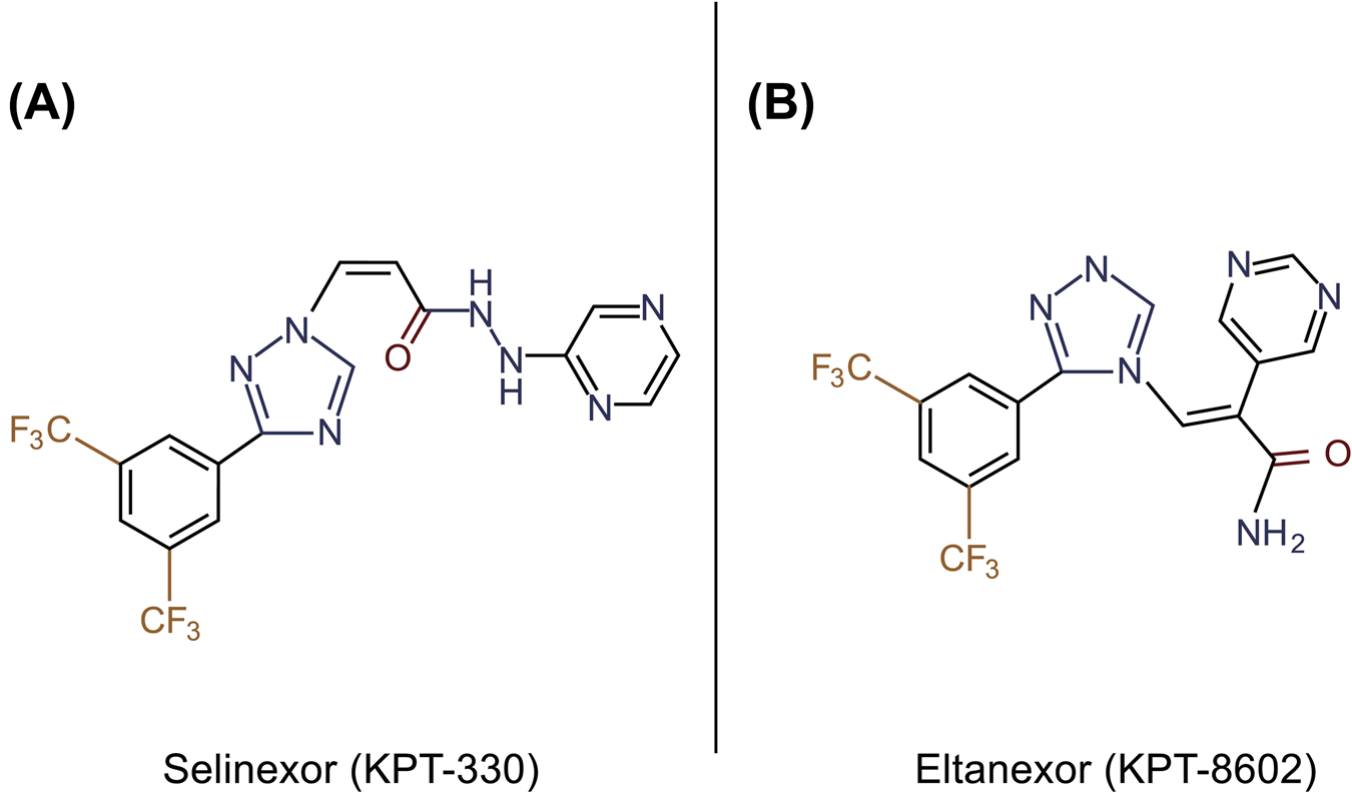
Chemical structures of the selective inhibitors of nuclear export (SINEs) selinexor and eltanexor. (A) Selinexor (KPT‐330). (B) Eltanexor (KPT‐8602).

SINE compounds inhibit XPO1 by forming slowly reversible covalent bonds with a critical cysteine residue in the NES‐binding cleft, thereby blocking NES‐dependent nuclear export and simultaneously perturbing multiple signalling pathways relevant to leukaemogenesis.^60^ First‐generation agents such as selinexor (KPT‐330) established the feasibility of targeting XPO1 in AML, whereas the second‐generation inhibitor eltanexor (KPT‐8602), developed through structure–activity optimisation, was designed to retain anti‐leukaemic potency while improving tolerability.^61^ More recent preclinical studies have further clarified the anti‐leukaemic effects of XPO1 inhibition in AML, including its downstream signalling consequences, its activity in molecularly defined XPO1‐dependent subsets, and its potential for rational combination strategies.^57^ The chemical structures of the two major SINE compounds discussed in this section, selinexor and eltanexor, are shown in Figure 4.

### 3.1 Selinexor (KPT‐330)

Selinexor is the first orally bioavailable XPO1 inhibitor to enter clinical development and obtain regulatory approval in oncology. By covalently binding to Cys528 in XPO1, selinexor blocks interaction of the transporter with NES‐bearing cargoes and promotes nuclear retention of multiple tumour suppressor proteins, including p53, FOXO3A and RB1, thereby restoring their transcriptional and cell‐cycle regulatory functions. Early studies showed that selinexor induces cell‐cycle arrest and apoptosis in a broad panel of AML cell lines and primary blasts and significantly reduces bone marrow leukaemic burden in NSG xenograft models, while relatively sparing normal haematopoietic stem and progenitor cells (HSPCs), suggesting a favourable therapeutic index.^62^

Building on these observations, Emdal et al. performed systematic phosphoproteomic profiling of primary AML samples and cell lines treated ex vivo with selinexor.^56^ In selinexor‐sensitive samples, phosphorylation of p53 pathway components was markedly increased, with upregulation of downstream cell‐cycle inhibitors such as p21 and concomitant dephosphorylation of proteins involved in proliferation and cell‐cycle progression. These data indicate that intact p53 function is an important prerequisite for robust selinexor responses. By contrast, in low‐responder or resistant samples, the AKT–FOXO3 pathway remained highly active and partially counteracted p53‐mediated pro‐apoptotic signalling; pharmacologic inhibition of AKT enhanced the cytotoxicity of selinexor. These findings indicate at a global signalling level that the anti‐leukaemic effects of selinexor in AML are closely linked to p53 pathway activation and suppression of survival signalling, thereby providing a mechanistic rationale for combination with PI3K/AKT pathway inhibitors.

In NPM1‐mutated AML, XPO1 inhibition is underpinned by particularly compelling biological rationale. Mutant NPM1 (NPM1c), and some NPM1‐fusion proteins, acquire a novel NES motif that drives aberrant cytoplasmic localisation in an XPO1‐dependent manner and maintains HOX/MEIS‐driven leukaemic transcriptional programs. Pianigiani et al. showed in NPM1‐mutated cell lines and patient‐derived models that sustained selinexor exposure leads to stable downregulation of HOXA9, HOXA10 and MEIS1 and induction of monocytic differentiation markers such as CD11b, whereas intermittent dosing produces only transient HOX suppression and limited differentiation.^51^ These findings suggest that, in NPM1‐mutated AML, sustained XPO1 inhibition is required to achieve durable repression of HOX/MEIS programs and induction of terminal differentiation.

Shimosato et al. extended these observations to rare NPM1‐fusion subtypes, including NPM1::MLF1 and NPM1::CCDC28A. Using murine bone marrow transduction models and immortalised cell lines, the authors demonstrated that these fusions also act as XPO1‐dependent transcriptional activators of HOX genes. Selinexor treatment reduced binding of NPM1‐fusion proteins to HOX promoters, downregulated HOX expression and impaired clonogenic growth, supporting a potential role for XPO1 inhibition in NPM1‐fusion‐driven AML.^63^

Selinexor has also been evaluated in combination with cytotoxic chemotherapy in murine AML models. In a genetically defined C57BL/6J AML model, selinexor (e.g., 15 mg/kg orally, three times per week) combined with cytarabine (200 mg/kg subcutaneously) prolonged median survival and reduced peripheral blood and bone marrow leukaemic burden more effectively than either agent alone. However, the combination exerted suppressive effects on both leukaemic and normal haematopoiesis, with marked reductions in LSK HSPCs and common lymphoid progenitors, and impaired long‐term engraftment in secondary transplantation assays. These results underscore the need to balance enhanced anti‐leukaemic activity with preservation of normal haematopoietic reserve when designing selinexor‐based combinations.^64^

### 3.2 Eltanexor (KPT‐8602)

Eltanexor (KPT‐8602) is a second‐generation SINE developed through structural optimisation of selinexor, with the goal of preserving anti‐leukaemic activity while improving tolerability. It retains high affinity for XPO1 and a similar covalent mechanism of action, but exhibits lower central nervous system exposure and a more favourable pharmacokinetic and tissue‐distribution profile, enabling more frequent dosing with potentially improved gastrointestinal tolerability.^65^ In high‐risk adult AML patient‐derived xenograft (PDX) models, Etchin et al. showed that eltanexor achieves deeper depletion of bone marrow human CD45^+^ leukaemic cells than selinexor in both complex karyotype (AML‐CK) and cytogenetically normal (AML‐CN) models and more effectively reduces the frequency of leukaemia‐initiating cells (LICs), while exerting relatively limited long‐term effects on normal haematopoietic reconstitution in NSG mice transplanted with human CD34^+^ HSPCs.^62^ These findings support eltanexor as a potentially more potent and better tolerated XPO1 inhibitor in AML, with relatively limited long‐term effects on normal haematopoietic reconstitution in preclinical models.

More recent work has identified molecular subsets in which eltanexor might have particularly strong disease‐modifying potential. Charles Cano et al. investigated AML with t(6;9)(p23;q34)/DEK::NUP214 and found that DEK::NUP214‐positive FKH‐1 cells are highly dependent on XPO1 for survival.^57^ Genetic ablation of XPO1 induced profound cell‐cycle arrest and apoptosis, whereas pharmacologic inhibition with eltanexor disrupted the nuclear and chromatin‐associated interaction between XPO1 and DEK::NUP214 and increased apoptosis with G2/M accumulation in primary DEK::NUP214‐positive samples. In PDX models, eltanexor treatment led to deep clearance of residual bone marrow disease and significantly prolonged survival. Mechanistically, chromatin immunoprecipitation and sequencing analyses showed that DEK::NUP214 and XPO1 co‐occupy promoters of HOXB8, HOXB9, PBX3 and other self‐renewal‐associated genes; eltanexor disrupted this chromatin‐associated complex, downregulated HOX/MEIS and stemness programs and impaired leukaemia self‐renewal.

Kaya et al. further validated XPO1 dependency in a multi‐omic cohort of 57 DEK::NUP214‐positive AML cases (including four with classical t(6;9)).^66^ Using large‐scale ex vivo drug‐sensitivity profiling against more than 500 compounds, they showed that XPO1 inhibitors (selinexor and eltanexor) rank among the most selective and potent hits in t(6;9) AML. RNA sequencing revealed marked upregulation of FOXC1 and HOXA/HOXB clusters, while CUT&RUN experiments demonstrated direct binding of DEK::NUP214 to FOXC1 and HOX promoters. XPO1 inhibition reduced DEK::NUP214 occupancy at these loci and downregulated associated transcriptional programs, confirming a critical DEK::NUP214–XPO1–HOX/MEIS axis in t(6;9) AML and providing a strong molecular rationale for eltanexor in this high‐risk subtype.

In NPM1‐mutated AML, Pianigiani et al. systematically evaluated eltanexor dosing schedules.^51^ In vitro, continuous exposure to 50 nM eltanexor for 5 days per week induced stable downregulation of HOXA9, HOXA10 and MEIS1 and robust upregulation of CD11b in NPM1‐mutated cell lines such as OCI‐AML3, whereas short or intermittent exposure produced only transient HOX/MEIS repression. In NPM1‐mutated PDX models, eltanexor (10 mg/kg, 5 days per week for 4 weeks) significantly reduced bone marrow human CD45^+^ leukaemic burden and prolonged survival, accompanied by increased CD11b expression and sustained HOX pathway suppression. These data indicate that sustained XPO1 inhibition is crucial for durable HOX cluster repression and induction of terminal differentiation in NPM1‐mutated AML and are consistent with observations from selinexor‐based studies.

In spliceosome‐mutated disease, Chaudhry et al. investigated SF3B1‐mutated high‐risk MDS/AML models, including conditional Sf3b1^K700E knock‐in mice.^50^ Eltanexor induced widespread nuclear retention of poly(A)^+^ RNA and aberrant alternative splicing, particularly in transcripts involved in apoptosis regulation and RNA processing. CRISPR‐based forward genetic screens identified BCL2 and BCL‐XL as key collaborative nodes, and in vivo experiments confirmed that eltanexor plus venetoclax preferentially depleted SF3B1‐mutated clones with acceptable systemic toxicity. These findings provide a strong preclinical rationale for a precision‐medicine strategy based on eltanexor plus BCL2 inhibition in SF3B1‐mutated MDS/AML.

### 3.3 Combination strategies: Evidence from preclinical models

Because XPO1 inhibition simultaneously affects the p53 pathway, BCL2 family members, MYC‐ and HOX/MEIS‐driven programs, and multiple stress‐response networks, rational combinations have become a major focus of translational development for SINE compounds in AML. Current preclinical studies have primarily explored combinations with BCL‐2 inhibitors, epigenetic and transcription‐modulating agents and metabolic modulators. An overview of key preclinical studies of XPO1 inhibition in AML, including disease models, molecular subtypes, combination partners and mechanistic insights, is summarised in Table 1.

**TABLE 1:** Representative preclinical studies of XPO1 inhibition in acute myeloid leukaemia.

Author (year)	SINE agent	AML models	Molecular features/subtypes	Combination partner(s)	Key mechanistic findings
Kojima et al. (2013)	KPT‐185	AML cell lines; primary AML blasts	Bulk AML; TP53WT versus TP53mut	–	XPO1 overexpression associated with adverse prognosis; KPT‐185 induces apoptosis preferentially in TP53WT AML.^73^
Etchin et al. (2016)	Selinexor	Adult primary AML; NSG AML PDX (CK, CN)	High‐risk adult AML; LIC‐enriched	–	Selinexor kills AML blasts and LICs while relatively sparing normal HSPCs; supports XPO1 as a therapeutic vulnerability.^74^
Etchin et al. (2017)	Eltanexor	AML cell lines; primary AML; AML PDX	High‐risk AML; LIC‐enriched	–	Second‐generation XPO1 inhibitor with improved tolerability; deeper depletion of LICs and stronger anti‐leukaemic activity.^62^
Emdal et al. (2022)	Selinexor	Primary AML samples ex vivo; AML cell lines	Heterogeneous AML; TP53 context	–	Phosphoproteomics shows selinexor response depends on p53 pathway activation; AKT–FOXO3 signalling modulates sensitivity.^56^
Pianigiani et al. (2022)	Eltanexor	OCI‐AML3; NPM1mut AML PDX	NPM1‐mutated AML	–	Sustained XPO1 inhibition continuously downregulates HOX/MEIS and induces CD11b^+^ differentiation; intermittent dosing fails.^51^
Shimosato et al. (2024)	Selinexor	Mouse BM transduction models; immortalised cells	NPM1::MLF1, NPM1::CCDC28A fusion AML‐like models	–	NPM1‐fusions act as XPO1‐dependent transcriptional activators of HOX genes; selinexor suppresses HOX and colony formation.^63^
Cano et al. (2025)	Eltanexor	FKH‐1; primary DEK::NUP214 AML; PDX	t(6;9)(p23;q34)/DEK::NUP214‐positive AML	–	DEK::NUP214 leukaemia is highly XPO1‐dependent; eltanexor reduces DEK::NUP214–XPO1 chromatin occupancy and HOXB/PBX programs.^57^
Kaya et al. (2025)	Selinexor, Eltanexor	57 primary AML samples; DEK::NUP214+ versus others	t(6;9)/DEK::NUP214 AML	–	Large‐scale drug screen shows XPO1i as top hits in t(6;9) AML; multi‐omics define DEK::NUP214 as XPO1‐dependent HOX activator.^66^
Chaudhry et al. (2024)	Eltanexor	SF3B1mut MDS/AML models; conditional Sf3b1K700E mice	SF3B1‐mutated high‐risk MDS/AML	Venetoclax	XPO1 inhibition causes RNA nuclear retention and aberrant splicing; eltanexor + venetoclax preferentially kill SF3B1mut cells.^50^
Ranganathan et al. (2016)	Selinexor	AML cell lines; primary AML; xenografts	Adult AML	Cytarabine; decitabine	XPO1 inhibition re‐nuclearises topo IIα and downregulates DNA‐repair/CHK1–WEE1 axes; synergises with cytotoxic chemotherapy.^75^
Fischer et al. (2020)	Selinexor, Eltanexor	AML cell lines (MOLM‐13, MV4‐11); DLBCL; AML xenografts	FLT3‐ITD+, KMT2A‐rearranged AML; B‐cell lymphomas	Venetoclax	SINE drugs reduce XPO1 and MCL1, restore p53 and enhance apoptosis; pronounced in vitro and in vivo synergy with venetoclax.^67^
Yu et al. (2022)	Selinexor, Eltanexor	AML cell lines; primary AML blasts	Bulk AML	Venetoclax	Venetoclax augments XPO1i‐induced DNA damage; XPO1i downregulate MYC, CHK1, WEE1, RAD51, RRM2 and venetoclax impairs repair.^68^
Long et al. (2023)	Selinexor	AML cell lines; primary AML cells	Adult AML; XPO1/eIF4Ehigh	Azacitidine	AZA + selinexor synergistically inhibit proliferation and induce apoptosis via coordinated XPO1, eIF4E and c‐MYC downregulation.^69^
Jiang et al. (2023)	Selinexor	AML cell lines; primary ND and R/R AML samples	VEN‐sensitive and VEN‐resistant AML	Venetoclax	VEN + SEL combination enhances apoptosis; suppresses glycolytic function and DNA‐replication genes (POLA2, POLD2, POLE2).^76^
Deng et al. (2025)	Selinexor	MLL‐r AML cell lines; ex vivo samples; MLL‐r PDX	MLL‐rearranged AML	Triptolide (TPL)	Low‐dose TPL + selinexor synergise via Rap1/Raf/MEK/ERK‐mediated MYC downregulation, MOMP and mitochondrial apoptosis.^70^
Wang et al. (2025)	Selinexor	Multiple AML/ALL lines; leukaemia xenografts	AML, MLL‐AF9 and other MYC‐driven leukaemias	JQ1 (BET inhibitor)	Selinexor + JQ1 dual‐target C‐MYC; combination shows strong synergy, cell‐cycle arrest and apoptosis, improved survival in vivo.^71^
Weiss et al. (2025)	Selinexor	AML cell lines; primary AML samples; xenografts	Adult AML	Atovaquone	Atovaquone + selinexor re‐shape STAT3 signalling (↑STAT3β/α ratio) and mitochondrial metabolism, enhancing pro‐apoptotic effects.^72^

Abbreviations: AML, acute myeloid leukaemia; LIC, leukaemia‐initiating cell; ND, newly diagnosed; R/R, relapsed/refractory; SINE, selective inhibitor of nuclear export.

Among BCL2‐directed combinations, Fischer et al. systematically assessed the interaction between venetoclax and SINE compounds (selinexor and eltanexor) in AML and diffuse large B‐cell lymphoma models.^67^ In AML cell lines such as MOLM‐13 and MV4‐11, dose‐response matrix analyses consistently demonstrated synergy, with combination treatment producing greater reductions in cell viability and higher Annexin V positivity than either agent alone. Mechanistic studies showed that SINE treatment downregulates XPO1 and MCL1, restores p53 activity, and sensitises mitochondria to apoptosis, while venetoclax further lowers the anti‐apoptotic threshold. Colony‐forming assays revealed markedly greater suppression of clonogenicity with the combination. In AML xenograft models, eltanexor (e.g., 7.5 mg/kg) plus venetoclax (25 mg/kg) more effectively reduced bone marrow and splenic human CD45^+^ leukaemic infiltrates and prolonged survival compared with monotherapy.

Yu et al. approached the combination from a DNA‐damage perspective, showing that selinexor monotherapy increases γH2AX levels, consistent with accumulation of DNA double‐strand breaks, and that addition of venetoclax further augments γH2AX while downregulating DNA repair proteins such as RAD51 and CHK1. Expression of RRM2 and WEE1 was also reduced, impairing the DNA‐damage response and lowering cellular tolerance to genotoxic stress. In AML models, the combination produced an additional 40%–50% reduction in cell viability relative to either single agent.^68^ In SF3B1‐mutated MDS/AML models, the aforementioned study by Chaudhry et al. demonstrated that eltanexor plus venetoclax more effectively suppresses SF3B1‐mutated clones than eltanexor alone, further supporting the concept that XPO1 inhibition plus BCL2 inhibition may represent a genotype‐informed rather than purely empirical combination strategy.

Combinations with epigenetic and transcriptional regulators have also shown promise. Long et al. reported that azacitidine plus selinexor synergistically inhibits proliferation and induces apoptosis in AML cell models.^69^ Mechanistically, azacitidine attenuates c‐MYC‐driven oncogenic transcriptional programs, whereas selinexor disrupts XPO1/eIF4E‐dependent nuclear export and translation of MYC and its target transcripts, resulting in multi‐layered suppression of the MYC axis. In KMT2A (MLL)‐rearranged AML, Deng et al. found that low‐dose triptolide combined with selinexor markedly enhances mitochondrial apoptosis. In cell lines and PDX models, this combination inhibited the Rap1/Raf/MEK/ERK signalling cascade, downregulated MYC, reduced mitochondrial membrane potential, increased reactive oxygen species (ROS) accumulation and elevated γH2AX, thereby amplifying DNA damage and triggering apoptosis.^70^ Wang et al. further showed that selinexor plus the BET inhibitor JQ1 exerts synergistic anti‐leukaemic effects in multiple AML cell lines and leukaemia xenografts, achieving >85% growth inhibition at relatively low doses with combination indices <1, more profound downregulation of C‐MYC, increased cleaved PARP and cell‐cycle arrest.^71^ In MLL‐AF9‐driven CDX/PDX models, selinexor plus JQ1 significantly reduced leukaemic infiltration in bone marrow, spleen and liver and extended survival. Collectively, these studies highlight that XPO1 inhibitors can cooperate with hypomethylating agents, BET inhibitors and other transcriptional modulators through convergent targeting of MYC and HOX/MEIS oncogenic axes.

In the realm of metabolic and other non‐canonical combinations, Weiss et al. reported that the antimalarial agent atovaquone synergises with selinexor in AML cell lines, primary samples and xenograft models.^72^ In vitro, the combination more effectively inhibits cell viability and induces apoptosis than either monotherapy. Mechanistic analyses indicated that atovaquone perturbs mitochondrial oxidative phosphorylation by inhibiting the electron transport chain, whereas selinexor blocks XPO1‐mediated nuclear export; together, they increased the STAT3β/α ratio and upregulated downstream targets such as SELL (CD62L), thereby reshaping STAT3 signalling and enhancing anti‐leukaemic effects. In xenograft models, atovaquone plus selinexor reduced bone marrow leukaemic infiltration and prolonged survival, and higher CD62L expression was associated with improved outcomes. These findings suggest that integrating XPO1 inhibition with agents that target energy metabolism may offer additional combination opportunities in relapsed or refractory AML.

Overall, current preclinical evidence indicates that specific molecular subsets, including NPM1‐mutated, t(6;9)/DEK::NUP214‐positive and SF3B1‐mutated disease, exhibit heightened dependency on XPO1 and may be particularly susceptible to XPO1 inhibition. In terms of combination strategies, regimens pairing XPO1 inhibitors with BCL‐2 inhibitors, hypomethylating agents, BET inhibitors or metabolic modulators have shown synergistic anti‐leukaemic activity across diverse AML models and are supported by plausible mechanistic rationales. Key questions for future translational work include how to maintain sufficiently strong and sustained XPO1 inhibition while controlling haematologic and systemic toxicities, and how to select optimal combination partners based on molecular subtype and signalling pathway dependencies.

## 4 CLINICAL STUDIES OF XPO1 INHIBITION IN AML

Although XPO1 inhibitors have shown clear anti‐leukaemic activity in diverse AML models, clinical translation in AML remains at an early stage.^76^ Available data are dominated by phase I/II trials of selinexor in relapsed/refractory (R/R) disease and by studies of the second‐generation inhibitor eltanexor in high‐risk MDSs with excess blasts.^77^, ^78^ Overall, selinexor monotherapy has produced modest response rates in R/R AML, and although combinations with intensive chemotherapy or low‐intensity regimens can increase initial remission rates, the balance between toxicity and survival benefit is variable and the optimal clinical niche for XPO1 inhibition remains to be defined. An overview of key clinical studies of XPO1 inhibition in AML and related high‐risk myeloid neoplasms, together with their trial registration numbers where available, is provided in Table 2.

**TABLE 2:** Clinical trials of XPO1 inhibitors in acute myeloid leukaemia and related high‐risk myeloid neoplasms.

Trial ID	Phase/design	Population	Regimen (XPO1 backbone)	Key efficacy outcomes	Key safety/notes
NCT01607892^80^	Phase I, dose‐escalation, single‐arm	95 patients with R/R AML (advanced haematologic malignancies; AML expansion cohort)	Selinexor monotherapy (16–70 mg/m^2^, 2–3 times per week)	ORR 14% (11/81 evaluable; CR/CRi/MLFS/PR); ∼31% had ≥50% BM blast reduction; median OS 2.7 months overall, 9.7 months in responders	Predominantly grade 1–2 GI and constitutional AEs; fatigue was main ≥grade 3 non‐haematologic AE; no formal DLT, RP2D 60 mg BIW
NCT02088541^79^	Phase II, randomised 2:1, open‐label (SOPRA)	≥60‐year R/R AML, selinexor versus physician's choice	Selinexor 60 mg BIW versus investigator's choice therapy	Median OS 3.2 versus 5.6 months (HR≈1.2, NS); CR/CRi rates low in both arms	Higher rates of grade ≥3 thrombocytopenia, febrile neutropenia, hyponatremia in selinexor arm
NCT02093403^84^	Phase I, dose‐escalation, single‐arm	25 adults with R/R AML or ≥60‐year untreated unfit AML	Decitabine 20 mg/m^2^ d1–10 + selinexor BIW (23–55 mg/m^2^; RP2D 60 mg flat dose BIW)	ORR 40% (CR/CRi/MLFS); responses seen in both newly diagnosed elderly and R/R cohorts	No protocol‐defined DLT; frequent grade ≥3 hyponatremia (≈68%), febrile neutropenia, sepsis, hypophosphatemia, pneumonia
NCT02573363^85^	Phase I, dose‐escalation with expansion	20 patients with newly diagnosed or R/R high‐risk AML	Selinexor (60→80 mg BIW) + HiDAC/Mitoxantrone (age‐adjusted)	ORR 70% (CR 50%, CRi 15%, PR 5%); 57% of responders proceeded to allo‐HSCT	No DLT; common AEs: febrile neutropenia (70%), diarrhoea, anorexia, electrolyte abnormalities; RP2D selinexor 80 mg BIW
NCT02299518^77^	Phase I, dose‐escalation, single‐arm	23 adults <60 years with R/R AML	Selinexor (30–55 mg/m^2^ BIW, d1,3,8,10,15) + MEC (mitoxantrone/etoposide/cytarabine)	ORR 43% (CR 26%, CRi 9%, MLFS 9%); 7/10 responders bridged to allo‐HSCT	DLT: hyponatremia at higher dose; common grade ≥3 AEs: febrile neutropenia, catheter infections, diarrhoea, hyponatremia, sepsis
NCT02416908^64^	Phase I/II, single‐arm	40 adults with R/R AML	Selinexor 60 mg (d1,5,10,12) + CLAG (cladribine/cytarabine/G‐CSF)	CR/CRi 45% (18/40); median EFS 6.1 months, median OS 7.8 months; ∼60% of patients proceeded to allo‐HSCT	30‐ and 60‐day mortality 2.5% and 7.5%; main ≥grade 3 AEs were infectious and GI; GI toxicity manageable with dose scheduling
NCT02649790^65^	Phase I/II, open‐label	20 patients with high‐risk MDS, HMA‐refractory (many MDS‐EB with 5%–19% blasts; MDS/AML borderline)	Eltanexor 10 or 20 mg QD d1–5 q28d (monotherapy)	In 15 evaluable patients, ORR 53.3% with mCR 46.7%; median OS ∼9.9 months	No DLT; common AEs: nausea, diarrhoea, decreased appetite, fatigue, neutropenia; generally manageable with dose modification

Abbreviations: allo‐HSCT, allogeneic haematopoietic stem cell transplantation; AML, acute myeloid leukaemia; BIW, twice weekly; CR, complete remission; CRi, complete remission with incomplete haematologic recovery; EFS, event‐free survival; HMA, hypomethylating agent; MDS, myelodysplastic syndrome; MDS‐EB, myelodysplastic syndrome with excess blasts; MLFS, morphologic leukaemia‐free state; ORR, overall response rate; OS, overall survival; QD, once daily; R/R, relapsed/refractory; RP2D, recommended phase II dose; XPO1i, XPO1 inhibitor.

### 4.1 Selinexor monotherapy in AML

In the randomised, open‐label phase II SOPRA trial (NCT02088541), Sweet et al. assigned patients aged ≥60 years with R/R AML in a 2:1 ratio to receive selinexor 60 mg twice weekly or investigator's choice of therapy (*n* = 118 vs. 57).^79^ Median overall survival (OS) was 3.2 months in the selinexor arm and 5.6 months in the physician's choice arm (HR ≈ 1.18, *p* = .422), with low rates of CR or CR with incomplete haematologic recovery (CRi) in both groups. Grade ≥3 thrombocytopenia, febrile neutropenia and hyponatremia were more frequent with selinexor. The authors concluded that, in an unselected, older R/R AML population, selinexor monotherapy confers limited anti‐leukaemic activity with a substantial toxicity burden and is better suited as a backbone for combination strategies rather than as standalone therapy.

In an earlier phase I dose‐escalation study (NCT01607892), Garzon et al. treated 95 patients with R/R AML with selinexor across a range of doses.^80^ Among 81 evaluable patients, the overall response rate (CR/CRi/marrow leukaemia‐free state (MLFS)/partial remission) was 14% (11/81), and approximately 31% achieved ≥50% reduction in bone marrow blasts. Median OS was 2.7 months in the overall cohort, whereas responders had a median OS approaching 9–10 months, indicating that a subset of high‐risk patients can achieve relatively durable disease control. Taken together, current monotherapy data suggest that selinexor has modest efficacy in heavily pretreated R/R AML and that its most promising role is likely within rationally designed combination regimens rather than as a single‐agent standard of care.

### 4.2 Selinexor combined with intensive chemotherapy

In the salvage setting for R/R AML, selinexor has been combined with intensive chemotherapy in several early‐phase trials. The phase II SAIL study (NCT02249091) evaluated selinexor plus standard ‘7 + 3’ (cytarabine plus idarubicin) in patients with R/R AML (*n* = 42).^81^ The initial selinexor dose was 40 mg/m^2^ twice weekly for 4 weeks; however, this schedule was associated with prolonged neutropenia, with febrile neutropenia and grade 3–4 diarrhoea occurring in 85% and 56% of patients, respectively. The regimen was subsequently amended to a fixed selinexor dose of 60 mg twice weekly for 3 weeks, which improved tolerability. In the overall cohort, the objective response rate (ORR; CR/CRi/MLFS) was 50%, and approximately one‐third of patients proceeded to allogeneic haematopoietic stem cell transplantation (allo‐HSCT). Median EFS, relapse‐free survival (RFS) and OS were 4.9, 17.7 and 8.2 months, respectively. These data suggest that selinexor plus 7 + 3 has meaningful salvage potential in selected R/R AML patients, but careful optimisation of dose and schedule is essential to balance efficacy with marrow suppression and gastrointestinal toxicity.

Bhatnagar et al. conducted a phase I dose‐escalation study (NCT02299518) of selinexor combined with mitoxantrone, etoposide and cytarabine (MEC) in adults <60 years with R/R AML (*n* = 23).^77^ The maximum tolerated dose of selinexor was 30 mg/m^2^, with dose‐limiting toxicity consisting primarily of grade 3–4 hyponatremia. The ORR was 43%, including a CR rate of 26%, indicating that this combination has clinically relevant salvage activity, although electrolyte disturbances and infection‐related toxicities require close monitoring and supportive care.

In newly diagnosed high‐risk or adverse‐risk AML, Sweet et al. evaluated selinexor combined with daunorubicin plus cytarabine induction chemotherapy in a phase I study (NCT02403310).^82^ At the recommended phase II dose of 80 mg selinexor twice weekly, the ORR was approximately 70%, with a CR rate of around 50%, demonstrating substantial anti‐leukaemic activity. However, grade ≥3 gastrointestinal adverse events and electrolyte abnormalities were common, underscoring the need for dose optimisation and proactive supportive management when integrating selinexor into frontline intensive regimens.

Importantly, a multicentre, open‐label, randomised phase II trial by Janssen et al. (Netherlands Trial Registry number NL5748 [NTR5902]) evaluated the addition of selinexor to standard ‘3 + 7’ induction in patients aged ≥65 years who were considered fit for intensive chemotherapy (*n* = 102; 51 per arm).^83^ In this study, the control arm achieved a CR/CRi rate of 80%, significantly higher than the 59% observed in the selinexor arm. Eighteen‐month EFS and OS were 45% and 58% in the control arm, compared with 26% and 33% in the selinexor arm, respectively. The selinexor arm also had higher rates of infection, sepsis and treatment‐related mortality, without improvement in measurable residual disease (MRD) negativity. These findings highlight that, in unselected older patients receiving frontline intensive therapy, simple addition of selinexor may not only fail to improve outcomes but can also worsen tolerability, arguing against its use as a universal first‐line intensification strategy.

### 4.3 Selinexor combined with low‐intensity therapy

In older patients or those who are ineligible for intensive chemotherapy, selinexor has been explored in combination with low‐intensity regimens. In a phase I dose‐escalation study (NCT02093403), Bhatnagar et al. combined selinexor with low‐dose decitabine (20 mg/m^2^) in adults with R/R AML or newly diagnosed patients aged ≥60 years who were unfit for intensive therapy (*n* = 25).^84^ No protocol‐defined dose‐limiting toxicity was observed, and the recommended phase II dose was established as selinexor 60 mg twice weekly. The ORR was 40% (CR/CRi/MLFS), indicating that this combination can induce remission in a subset of high‐risk patients. However, grade ≥3 hyponatremia was frequent (approximately 68%), alongside high rates of febrile neutropenia, sepsis, hypophosphatemia and pneumonia. Shortening the duration of selinexor administration and switching to a flat‐dose schedule improved tolerability to some extent. Overall, selinexor combined with low‐intensity hypomethylating therapy appears feasible in selected difficult‐to‐treat patients, but its use is constrained by electrolyte disturbances and infection‐related toxicities.

Beyond conventional clinical endpoints, integration of clonal dynamics and MRD assessment may help refine patient selection for XPO1 inhibitor‐based combinations. In a correlative subanalysis of the SAIL trial, Klement et al. examined clonal evolution in 15 patients with R/R AML treated with selinexor plus chemotherapy.^81^ Variant allele frequencies of clones harbouring FLT3, SF3B1 and TP53 mutations declined substantially after treatment, whereas those involving age‐related clonal haematopoiesis genes such as DNMT3A, ASXL1 and TET2 remained stable or increased slightly. Median survival differences between these patterns had not emerged definitively within the available follow‐up. Nonetheless, these observations suggest that incorporating clonal and MRD dynamics into future trials could provide additional insights into which patients derive durable benefit from XPO1 inhibitor‐containing regimens, and may help establish clinically relevant pharmacodynamic and early resistance biomarkers for treatment monitoring.

### 4.4 Clinical exploration of the second‐generation inhibitor eltanexor

Eltanexor is a second‐generation XPO1 inhibitor designed to achieve lower central nervous system exposure and improved gastrointestinal tolerability compared with selinexor, thereby enabling more frequent or prolonged dosing. Although large prospective trials in AML are not yet available, studies in high‐risk MDS with excess blasts offer important clues. In a phase I/II open‐label trial (NCT02649790), Lee et al. evaluated oral eltanexor monotherapy in patients with high‐risk MDS that was primary refractory or relapsed after hypomethylating agents; many had 5%–19% bone marrow blasts, approximating the MDS/AML boundary in the WHO 2022 classification.^65^ Among 15 evaluable patients treated with eltanexor 10 or 20 mg once daily on days 1–5 of a 28‐day cycle, the ORR was 53.3%, with a marrow complete remission (mCR) rate of 46.7% and median OS of approximately 9.9 months. No dose‐limiting toxicity was observed. The most common treatment‐related adverse events were nausea, diarrhoea, decreased appetite, fatigue and neutropenia, most of which were manageable with dose modification and supportive care.

When viewed together with extensive preclinical data in NPM1‐mutated, t(6;9)/DEK::NUP214‐positive and SF3B1‐mutated MDS/AML, these findings suggest that eltanexor is more likely to find its niche in AML through ‘molecularly selected plus sustained inhibition’ strategies rather than by replicating selinexor schedules in unselected populations. Prospective data in typical AML cohorts are still scarce; however, the safety profile and preliminary efficacy observed in high‐risk MDS with excess blasts provide a rationale for designing trials that span the MDS–AML continuum and focus on genetically and biologically defined subsets.

## 5 RISKS AND CHALLENGES OF XPO1 INHIBITION

Although XPO1 inhibitors have demonstrated anti‐leukaemic activity in preclinical models and early‐phase clinical trials, both the durability of responses and the safety profile remain major constraints on their broader use. In a cohort of patients with poor‐risk newly diagnosed AML treated with selinexor plus 7 + 3, Sweet et al. reported a CR/CRi rate of approximately 53% but a median OS of only approximately 10.3 months, indicating that long‐term outcomes remain limited even in the context of intensive chemotherapy.^82^ In a phase I study combining selinexor with high‐dose cytarabine and mitoxantrone, the overall response rate approached 70%; however, a proportion of patients still experienced rapid progression or early relapse.^85^ Mechanistic work suggests that leukaemic cells can attenuate the effects of XPO1 inhibition through multiple adaptive routes, including upregulation of XPO1 itself, remodelling of the broader nucleocytoplasmic transport machinery and activation of stress‐response pathways such as heat‐shock proteins, lipid metabolism and oxidative phosphorylation.^86^, ^87^ In some models, TP53 functional status and aberrant activation of the c‐MYC/E2F axis have also been linked to reduced sensitivity and acquired resistance.^88^, ^89^, ^90^ Collectively, these findings suggest that, at the current stage of development, XPO1 inhibitors are more likely to be effective as mechanistically informed components of combination or sequential regimens designed to deepen and prolong responses to existing therapies, rather than as monotherapy intended to provide durable long‐term disease control.

From a safety perspective, the toxicity profile of XPO1 inhibitors is closely related to their broad suppression of nuclear export in normal haematopoietic and non‐haematopoietic tissues. In phase I trials combining selinexor with intensive chemotherapy, the most common grade 3–4 non‐haematologic adverse events included febrile neutropenia (up to approximately 67%), diarrhoea (approximately 29%), hyponatremia (approximately 29%) and sepsis (approximately 14%).^82^ In regimens incorporating high‐dose cytarabine and mitoxantrone, the incidence of febrile neutropenia was approximately 70%, with diarrhoea (approximately 40%), anorexia (approximately 30%), electrolyte disturbances (approximately 30%) and fatigue and nausea/vomiting (approximately 25%) also frequently reported.^85^ A large real‐world pharmacovigilance analysis of the FAERS database identified 4392 adverse event reports in which selinexor was listed as a suspected drug. Gastrointestinal toxicities accounted for approximately 23.1% of reports, haematologic abnormalities such as thrombocytopenia and anaemia for approximately 6.9%, and non‐specific symptoms including fatigue, weight loss and dizziness for approximately 12.9%. Notably, 55.3% of events were associated with hospitalisation and 28.9% with death, underscoring the non‐trivial risk of serious outcomes.^91^ Preclinical work has further shown that selinexor‐associated thrombocytopenia is related to inhibition of thrombopoietin signalling in early megakaryocytic progenitors, consistent with a ‘target‐related’ on‐mechanism toxicity.^92^ In patients with AML, who often have compromised bone marrow reserve and poor nutritional status at baseline, these toxicities are accentuated and frequently necessitate dose reductions, treatment delays or interruptions, together with intensive supportive care. This, in turn, narrows the practical treatment window and constrains the intensity with which XPO1 inhibitors can be combined with other myelosuppressive agents.

In response to these limitations, second‐generation XPO1 inhibitors such as eltanexor have been designed with altered pharmacokinetic and tissue‐distribution properties to reduce central nervous system and systemic exposure and thereby improve tolerability. In a phase I/II trial in high‐risk, HMA‐refractory MDS, many patients had 5%–19% bone marrow blasts, approximating the MDS/AML boundary, and no formal dose‐limiting toxicities were observed. The overall incidence of grade 3–4 adverse events was also lower than that reported for selinexor in AML cohorts. Nevertheless, approximately 35% of patients required treatment delays and approximately 40% needed dose reductions owing to toxicity, indicating that the burden of adverse events, although mitigated, remains clinically relevant.^65^ Consequently, a key challenge for the AML field is to maintain pharmacodynamically effective and sufficiently sustained XPO1 inhibition while controlling haematologic and systemic toxicities. Achieving this goal will require optimisation of dosing schedules, proactive toxicity management and the development of biomarkers that can predict both efficacy and intolerance, thereby enabling rational selection of patients and personalised application of XPO1 inhibitors in specific clinical contexts.

## 6 TRANSLATIONAL PERSPECTIVES AND DISCUSSION

AML is characterised by high rates of relapse and treatment resistance, and long‐term remission and survival remain particularly limited in older patients and those with multiple comorbidities.^93^, ^94^ Venetoclax‐based low‐intensity regimens have become a major therapeutic option for older and unfit patients and, in both randomised trials and real‐world cohorts, have clearly improved initial remission rates and overall survival.^95^ Nonetheless, median EFS or RFS generally remains in the range of 8–10 months, and relapse is frequently accompanied by multiclonal evolution and acquired resistance, leaving very few effective salvage options.^96^, ^97^ Introducing agents with genuinely novel mechanisms of action into this heavily pretreated space has therefore become a central challenge in contemporary AML practice.

Against this backdrop, XPO1 inhibition, typified by selinexor, offers a mechanistically distinct approach that aims to reprogram leukaemic signalling at the level of nucleocytoplasmic transport. Selinexor is currently approved for relapsed/refractory multiple myeloma and diffuse large B‐cell lymphoma, whereas its role in AML is still being defined.^22^, ^98^ By covalently targeting Cys528 in XPO1 and blocking aberrant nuclear export of tumour suppressor proteins and oncogenic transcriptional regulators, XPO1 inhibitors have a strong biological rationale in NPM1‐mutated or ‐fusion AML, DEK::NUP214‐positive disease, spliceosome‐mutated subsets and certain therapy‐resistant clones.^66^, ^99^ Preclinical studies have consistently shown that XPO1 inhibition reduces leukaemic burden, targets LICs and synergises with hypomethylating agents, BCL‐2 inhibitors and BET inhibitors across diverse AML models, providing a solid mechanistic foundation for clinical exploration in high‐risk and relapsed/refractory populations. However, clinical data in AML remain limited and are still derived largely from small, early‐phase or single‐arm studies, whereas controlled evidence in clearly defined subgroups remains scarce.

One of the main barriers to broader implementation of XPO1 inhibitors in AML is the narrow therapeutic window imposed by their toxicity profile. First‐generation agents such as selinexor have demonstrated anti‐leukaemic activity in phase I/II trials, but this activity is accompanied by substantial haematologic and non‐haematologic toxicities, including thrombocytopenia, neutropenia, increased infection risk and gastrointestinal adverse events such as nausea, anorexia, weight loss and hyponatremia. In a patient population with already compromised bone marrow reserve and poor nutritional status, these toxicities are often magnified and frequently necessitate dose reductions, treatment delays or interruptions, along with intensive supportive care. This reality compresses the practical window for sustained treatment and limits the intensity with which XPO1 inhibitors can be combined with other myelosuppressive therapies. This may also help explain why selinexor has not successfully moved into earlier lines of therapy in AML: in a randomised trial, adding selinexor to frontline 3 + 7 in older patients eligible for intensive treatment failed to improve outcomes and even showed signs of inferiority relative to standard therapy in an unselected population. Second‐generation agents such as eltanexor, designed with altered pharmacokinetics and tissue distribution to reduce central nervous system and systemic exposure, have shown a more favourable tolerability profile in high‐risk MDS with excess blasts, but their efficacy and safety in typical AML cohorts still require rigorous, systematic evaluation.

These considerations suggest that the clinical value of XPO1 inhibition is unlikely to lie in providing another broadly applicable ‘one‐size‐fits‐all’ targeted therapy for AML. Instead, XPO1 inhibitors may be best deployed as components of carefully selected combination or sequential strategies in biologically defined settings and specific treatment windows. Future work could focus on: (1) molecular subtypes with putative high XPO1 dependency, such as NPM1‐mutated or NPM1‐fusion AML, t(6;9)/DEK::NUP214‐positive disease and spliceosome‐mutated subsets; (2) patients who fail venetoclax‐azacitidine or related regimens yet retain indications for intensive salvage therapy or allogeneic transplantation; and (3) individuals with persistent MRD or high‐risk clonal evolution signals, in whom XPO1 inhibition may be used to deepen responses or delay progression when integrated into risk‐adapted pathways. In these contexts, XPO1 inhibitors would function as targeted ‘add‐ons’ to existing backbones rather than as standalone backbone therapies.

Within this translational framework, the therapeutic implications of XPO1 mutations also warrant more explicit consideration. Unlike NPM1 mutations or DEK::NUP214 rearrangements, naturally occurring XPO1 mutations do not currently define a major molecular subtype in AML and appear to be relatively uncommon.^20^ Nevertheless, such mutations may still be biologically informative, because alterations in the NES‐binding region or related structural elements could influence cargo recognition, export selectivity and, potentially, the degree of leukaemic dependency on XPO1‐mediated nuclear export.^100^ From a therapeutic perspective, however, current evidence remains insufficient to support XPO1 mutation status as an established biomarker for selecting selinexor‐ or eltanexor‐based therapy in AML. At present, these lesions are better regarded as mechanistic clues that may help refine future studies of XPO1 dependency, subtype‐specific vulnerability and treatment adaptation, rather than as clinically actionable biomarkers in routine practice. Importantly, naturally occurring XPO1 mutations should also be distinguished from experimental alteration of the drug‐binding residue Cys528, which primarily provides on‐target validation and resistance‐related evidence for SINE compounds rather than reflecting a recurrent genomic feature of AML.^101^

More broadly, for the clinical development of XPO1 inhibitors in AML, biomarkers should be conceptualised in at least three complementary categories: predictive biomarkers, pharmacodynamic biomarkers and resistance biomarkers.^18^ Predictive biomarkers are needed to identify patient subsets most likely to benefit before treatment initiation and may include molecular contexts associated with heightened XPO1 dependency, such as NPM1‐mutated or NPM1‐fusion AML, t(6;9)/DEK::NUP214‐positive disease, SF3B1‐mutated myeloid neoplasms and, in some settings, TP53‐intact signalling states.^51^ Pharmacodynamic biomarkers are needed to confirm target engagement and biologic response during therapy and may include nuclear relocalisation of XPO1 cargoes, suppression of HOX/MEIS‐driven transcriptional programs, induction of differentiation markers such as CD11b, activation of p53/p21‐associated signalling and dynamic changes in MRD or mutation burden. Resistance biomarkers, in turn, may help explain primary refractoriness or acquired treatment failure and could include TP53 dysfunction, persistent AKT–FOXO3 or MYC/E2F signalling, adaptive remodelling of nucleocytoplasmic transport and stress‐response pathways, and clonal persistence or evolution under treatment pressure.^102^, ^103^ Prospectively integrating these three biomarker layers into future trials may improve patient selection, on‐treatment monitoring and relapse surveillance, and thereby facilitate a more precise clinical positioning of XPO1‐targeted strategies in AML.

At the same time, development of combination regimens should move from empirical stacking towards mechanism‐based integration. The emerging interplay between XPO1 inhibition and BCL2 dependency, MYC/E2F‐driven transcription, DNA‐damage response pathways and metabolic reprogramming provides a rationale for designing focused combinations at doses that are pharmacodynamically active yet tolerable. Trials that prioritise MRD dynamics, clonal evolution and durable survival endpoints, rather than short‐term response rates alone, will be crucial to identifying genuinely beneficial strategies. In parallel, biomarker development is essential. Rather than relying only on broad molecular annotation, future biomarker frameworks should distinguish predictive, pharmacodynamic and resistance biomarkers. Molecular signatures that capture XPO1 dependency, potentially incorporating NPM1, DEK::NUP214, SF3B1 and other lesions, as well as pharmacokinetic and pharmacodynamic markers of target engagement, will be needed to predict both efficacy and intolerance and to support individualised dosing and patient selection.

Overall, current data support XPO1 as a biologically compelling and clinically promising target in AML, yet widespread adoption awaits stronger evidence. XPO1 inhibitors are unlikely to serve as universal solutions for all relapsed/refractory cases, but they should not be dismissed as merely toxic or marginal options. The key clinical questions are whether, in clearly defined subgroups and treatment settings, appropriately dosed and rationally combined XPO1 inhibitors can meaningfully increase the probability of remission reinduction, expand opportunities for allogeneic transplantation or delay expansion of high‐risk clones while keeping incremental toxicity and economic burden within acceptable limits. Addressing these questions will require large, multicentre, randomised trials grounded in molecular stratification, complemented by long‐term follow‐up and real‐world data. Only through such integrative efforts can the precise place of XPO1‐targeted strategies within the evolving therapeutic landscape of AML be determined.

## AUTHOR CONTRIBUTIONS

Yifan Liu performed the literature review and drafted the manuscript. Weidong Ding and Xiaoya Yun critically reviewed and revised the manuscript. Suxiao Li contributed to the interpretation and integration of preclinical and clinical data. Hui Liu provided overall conceptual guidance, supervised the work and critically revised the manuscript.

## CONFLICT OF INTEREST STATEMENT

The authors declare no conflicts of interest.

## Data Availability

No data were generated for the research described in the article.
